# Procedural and Intermediate-term Results of the Electroanatomical-guided Cardioneuroablation for the Treatment of Supra-Hisian Second- or Advanced-degree Atrioventricular Block: the PIRECNA multicentre registry

**DOI:** 10.1093/europace/euae164

**Published:** 2024-07-02

**Authors:** Tolga Aksu, Roman Piotrowski, Roderick Tung, Tom De Potter, Timothy M Markman, Jeanne du Fay de Lavallaz, Roin Rekvava, Daniel Alyesh, Jacqueline E Joza, Patrick Badertscher, Duc H Do, Jason S Bradfield, Gaurav Upadhyay, Nitesh Sood, Parikshit S Sharma, Tumer Erdem Guler, Enes Elvin Gul, Vineet Kumar, Buelent Koektuerk, Alexander Romeno Janner Dal Forno, Christopher E Woods, Moshe Rav-Acha, Chiara Valeriano, Andres Enriquez, Sri Sundaram, Michael Glikson, Andre d’Avila, Kalyanam Shivkumar, Piotr Kulakowski, Henry D Huang

**Affiliations:** Department of Cardiology, Yeditepe University Hospital, Icerenkoy Mah. Hastahane Sok. 4, 34752, Istanbul, Turkey; Centre of Postgraduate Medical Education, Department of Cardiology, Grochowski Hospital, 01-809, Warsaw, Poland; The University of Arizona College of Medicine-Phoenix, Department of Cardiology, Banner University Medical Center, Phoenix, AZ 85004, USA; Cardiovascular Center, OLV Hospital, Aalst, 9300, Belgium; Division of Cardiology, Perelman School of Medicine, University of Pennsylvania, Philadelphia, PA 19104, USA; Department of Cardiology, University Hospital Basel, Petersgraben 4, 4031 Basel, Switzerland; Department of Rhythm Management and Electrophysiology, American Hospital Tbilisi, Tbilisi 01102, Georgia; South Denver Cardiology Associates, Littleton, CO 80120, USA; Department of Cardiology, McGill University, Montreal, QC, H3A 0G4, Canada; Department of Cardiology, University Hospital Basel, Petersgraben 4, 4031 Basel, Switzerland; UCLA Cardiac Arrhythmia Center, Ronald Reagan UCLA Medical Center, 100 Medical Plaza, Suite 660, Los Angeles, CA 90095, USA; UCLA Cardiac Arrhythmia Center, Ronald Reagan UCLA Medical Center, 100 Medical Plaza, Suite 660, Los Angeles, CA 90095, USA; Section of Cardiology, Center for Arrhythmia Care, University of Chicago Medicine, Chicago, IL 60637, USA; Department of Cardiology, Southcoast Health, Fall River, MA 02720, USA; Division of Cardiology, Rush University Medical Center, 1717 West Congress Parkway, Chicago, IL 60612, USA; Division of Cardiology, Kocaeli State Hospital, Kocaeli, 41060, Turkey; Division of Cardiology, Atlas University, Medicine Hospital, Istanbul, 34203, Turkey; Inova Medical Group, Arrhythmia 8081 Innovation Park Dr 602, Fairfax, VA 22031, USA; Department of Cardiology, Witten/Herdecke University, Witten, D-58455, Germany; Department of Cardiology and Electrophysiology, Sana Clinics Düsseldorf, Düsseldorf, 40625, Germany; Department of Cardiology, Hospital SOS Cardio, Florianópolis, SC, 88030-000, Brazil; Department of Cardiology, California Pacific Medical Center, San Francisco, CA 94110, USA; Jesselson Integrated Heart Center, Shaare Zedek Medical Center and Hebrew University Faculty of Medicine, PO Box 3235, Jerusalem 9103102, Israel; Cardiovascular Center, OLV Hospital, Aalst, 9300, Belgium; Division of Cardiology, Perelman School of Medicine, University of Pennsylvania, Philadelphia, PA 19104, USA; Division of Cardiology, Queen’s University, Kingston, ON K7l 3N6, Canada; South Denver Cardiology Associates, Littleton, CO 80120, USA; Jesselson Integrated Heart Center, Shaare Zedek Medical Center and Hebrew University Faculty of Medicine, PO Box 3235, Jerusalem 9103102, Israel; Beth Israel Deaconess Medical Center, Harvard Thorndike Electrophysiology Institute, Boston, MA 02215, USA; UCLA Cardiac Arrhythmia Center, Ronald Reagan UCLA Medical Center, 100 Medical Plaza, Suite 660, Los Angeles, CA 90095, USA; Centre of Postgraduate Medical Education, Department of Cardiology, Grochowski Hospital, 01-809, Warsaw, Poland; Division of Cardiology, Rush University Medical Center, 1717 West Congress Parkway, Chicago, IL 60612, USA

**Keywords:** Ablation, Atrioventricular block, Bradycardia, Ganglionated plexus, Syncope

## Abstract

**Aims:**

Prior case series showed promising results for cardioneuroablation in patients with vagally induced atrioventricular blocks (VAVBs). We aimed to examine the acute procedural characteristics and intermediate-term outcomes of electroanatomical-guided cardioneuroablation (EACNA) in patients with VAVB.

**Methods and results:**

This international multicentre retrospective registry included data collected from 20 centres. Patients presenting with symptomatic paroxysmal or persistent VAVB were included in the study. All patients underwent EACNA. Procedural success was defined by the acute reversal of atrioventricular blocks (AVBs) and complete abolition of atropine response. The primary outcome was occurrence of syncope and daytime second- or advanced-degree AVB on serial prolonged electrocardiogram monitoring during follow-up. A total of 130 patients underwent EACNA. Acute procedural success was achieved in 96.2% of the cases. During a median follow-up of 300 days (150, 496), the primary outcome occurred in 17/125 (14%) cases with acute procedural success (recurrence of AVB in 9 and new syncope in 8 cases). Operator experience and use of extracardiac vagal stimulation were similar for patients with and without primary outcomes. A history of atrial fibrillation, hypertension, and coronary artery disease was associated with a higher primary outcome occurrence. Only four patients with primary outcome required pacemaker placement during follow-up.

**Conclusion:**

This is the largest multicentre study demonstrating the feasibility of EACNA with encouraging intermediate-term outcomes in selected patients with VAVB. Studies investigating the effect on burden of daytime symptoms caused by the AVB are required to confirm these findings.

## Graphical Abstract

**Graphical Abstract:**
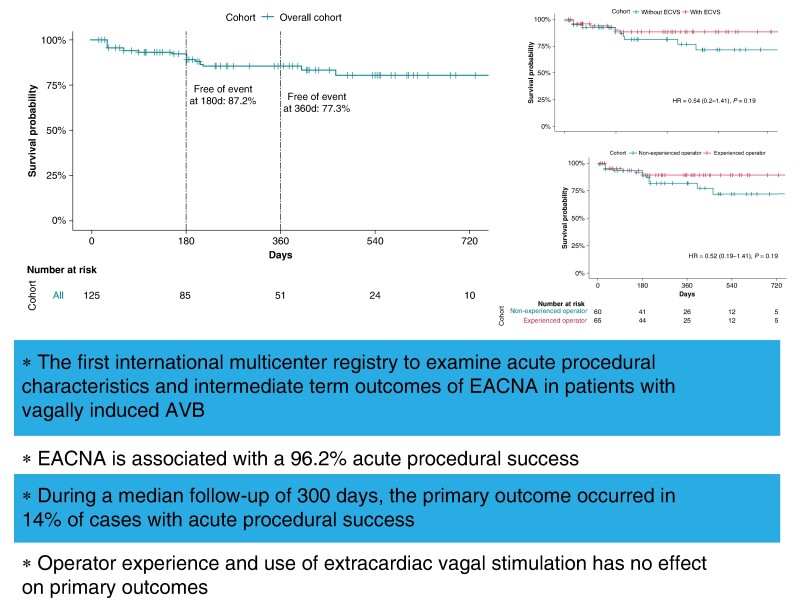

**What’s new?**

- Cardioneuroablation may improve patient outcomes and be an alternative to cardiac pacing in highly selected patients with functional atrioventricular blocks (AVBs).
- In 130 patients with functional AVBs, mainly bi-atrial cardioneuroablation was associated with 96.2% acute procedural success and 77.3% event-free survival.
- Survival analysis of the overall cohort with successful acute ablation did not show differences in event-free survival depending on the use of extracardiac vagal stimulation or operator experience.

## Introduction

A marked activation of the parasympathetic tone may cause vagally induced (extrinsic) atrioventricular blocks (VAVBs).^1,2^ These forms of bradyarrhythmias typically occur as paroxysmal episodes and are termed VAVB given the absence of an intrinsic abnormality within the atrioventricular (AV) conduction system.^1,2^ Patients with VAVBs may have associated symptoms even in the absence of syncope;^2^ however, it is often difficult to prove a causal relationship. Prolonged electrocardiogram (ECG) monitoring, evaluation of the AV response to atropine and exercise, and at times a detailed electrophysiological study (EPS) are required.

Prior case series and one small single-centre cohort study demonstrated the potential therapeutic role of ganglionated plexi (GPs) modulation by endocardial radiofrequency catheter ablation, also called cardioneuroablation (CNA), for the prevention of VAVB.^3–12^ However, to date, no multicentre study has evaluated the role of CNA in patients with VAVB. The present retrospective registry aims to examine acute procedural characteristics and medium-term outcomes of CNA in patients with VAVB.

## Methods

The data that support the findings of this registry are available from the corresponding authors upon reasonable request.

### Study design

The present multicentre retrospective international registry was designed to investigate outcomes associated with electroanatomical-guided CNA (EACNA) for patients with symptomatic VAVB. The registry was performed in accordance with the principles of the Declaration of Helsinki. All participants provided written informed consent for the ablation procedure. The collection of data was approved by the institutional review boards of the coordinating centre.

### Patient population

Patients undergoing EACNA for symptomatic VAVB in 20 international centres between 2021 and 2023 were included in the present study. All patients underwent detailed stepwise evaluation, including history, careful analysis of ECGs, prolonged ECG monitoring (Holter, event recorder, in-hospital telemetry, or implantable loop recorder), a physician-supervised exercise response test, and an invasive EPS to exclude intra-/infra-Hisian AVB, with bolus of intravenous atropine in patients presenting with persistent AVBs.

To define paroxysmal and persistent resting AVBs, two successive resting ECGs at least 24 h apart were evaluated in all cases. Patients demonstrating 1:1 AV conduction on at least one ECG were grouped as paroxysmal AVB, while a persistent resting AVB group was defined as having documentation of second- or advanced-degree AVB on at least two successive resting ECGs. The AV conduction disturbances of patients who were included in the study were classified as follows: (i) second-degree AVB: (a) Mobitz I second-degree AVB—progressive PR interval prolongation with each beat until a P wave is not conducted and (b) 2:1 AVB—one conducted P wave for each P wave blocked, and (ii) advanced AVB—two or more consecutive P waves are blocked. Only symptomatic patients, who had daytime second- or advanced-degree AVB episodes, were included in the study. Patients who had only nocturnal AVB were excluded. Cause–effect correlation between symptoms (i.e. fatigue, irritability, lassitude, inability to concentrate, lack of interest, forgetfulness, feeling of warmth, lightheadedness, dizziness, nausea, sweating, or syncope) and bradycardia was done by continuous electrocardiographic monitoring because bradycardia was intermittent in nature. All patients provided written informed consent for the procedure. The patients were informed that the CNA is a relatively new treatment method not yet recommended by the current guidelines.

The details of the inclusion and exclusion criteria for diagnosing VAVB cases are provided in *Table 1*.

**Table 1:** Inclusion and exclusion criteria

		Inclusion criteria	Exclusion criteria
All cases	History	Symptomatic bradycardia At least 1 syncope episode during the preceding 12 months or minimum of 2 pre-syncopal events during preceding 12 months	Use of negative chronotropic or dromotropic drugs Typical history for vasovagal syncope
	Laboratory assessment	Normal thyroid function tests	Thyroid function abnormality
	If suspicion of obstructive sleep apnoea	Negative polysomnogram	Positive polysomnogram
	Atropine response^a^	Positive	Negative
Paroxysmal AVB cases	Baseline ECG	Normal PR interval or first-degree AVB	
	Prolonged ECG monitoring	PR prolongation before second- or third-degree AVB episode	Constant PR before second- or third-degree AVB episode
		Decrease in sinus rate during or just prior to second- or third-degree AVB episode	Increase in sinus rate during second- or third-degree AVB episode
		Resolution of AVB with an increase in sinus rate	Resolution of AVB with a constant sinus rate
Persistent AVB cases	Baseline ECG	Mobitz Type I second- or advanced-degree AVB	Mobitz Type II second degree
	Atropine response^a^	Positive	Negative
	Electrophysiological study	Baseline H–V interval <55 ms	Baseline H–V interval ≥55 ms
		Supra-Hisian AVB	Intra/infra-Hisian AVB by atrial pacing at rates at 150 bpm or less

AVB, atrioventricular block; ECG, electrocardiogram.

^a^See text for details.

### Atropine response test

An atropine response test was performed in all cases, and only patients demonstrating a positive response were included in the present analysis. The test was carried out after a 4 h fasting with a maximum 0.04 mg/kg intravenous atropine sulphate, at least 24 h before the procedure. In patients with persistent AVB, return of 1:1 AV conduction or conversion from second- or third-degree to first-degree AVB was accepted as a positive response. In patients with paroxysmal AVB, demonstration of a >25% decrease in the PR interval (or not lengthened despite sinus rhythm acceleration) was defined as a positive response.

Post-atropine response was also used as a targeted clinical endpoint at end of the CNA procedure.

### Electroanatomical-guided cardioneuroablation

Ablation procedures were conducted under deep sedation or general anaesthesia. The use of depolarizing and non-depolarizing neuromuscular-blocking agents and anaesthetics causing sympathetic overactivity was avoided to facilitate evaluation of vagal response during radiofrequency energy delivery. For patients with persistent AVB or paroxysmal second-degree AVB with indeterminate level based on prior ECG data, a two-catheter conduction study was performed to measure AH and HV intervals and evaluate site of AVB through performance of straight atrial pacing at progressively shorter cycle lengths. In all cases, the right atrium (RA), the superior vena cava (SVC), the inferior vena cava, and the coronary sinus were mapped using an ablation or a mapping catheter. After RA mapping, transseptal puncture was performed. Before or immediately following transseptal access to the left atrium (LA), patients were anticoagulated with intravenous heparin to maintain an activated clotting time >300–350 s. Next, the LA, pulmonary veins (PVs), and left atrial appendage were mapped using an ablation or a mapping catheter. Bipolar endocardial electrograms were categorized as fractionated if they demonstrated three or more deflections or as normal atrial electrograms if they had less than three deflections. Fractionated electrograms in the regions known to be consistent with probable localization of GPs were identified and targeted using the three-dimensional (3D) mapping system. Intracardiac echocardiography (Acuson SC2000, Siemens, Germany; AcuNav™ Ultrasound Catheter, Biosense Webster, USA) and computed tomography were also used and served for identification of presumed GP areas based on operator discretion.

The following sites were ablated (number and order at discretion of the operator): the superior left atrial GP on the superoposterior surface of the LA, the Marshall tract GP on the endocardial aspect of the vein of Marshall, the inferior left atrial GP on the posterolateral surface of the LA, the posteromedial left atrial GP (PMLGP) between coronary sinus ostium and lower part of the LA, the superior right atrial GP (RSGP) between the SVC and the right superior PV, and the inferior right atrial GP (RIGP) adjacent to the interatrial groove.^13,14^

Standard irrigated-tip ablation catheters (Therapy Coolflex, FlexAbility, or TactiCath, St Jude Medical Inc., St Paul, MN, USA or Navistar, Thermocool, or Thermocool Smarttouch catheter, Biosense Webster Inc., Diamond Bar, CA, USA) were used in all radiofrequency ablation procedures.

Five patients underwent CNA only in the RA based on operator preference, and fractionated sites in the following areas were targeted and ablated: RSGP, RIGP, and PMLGP.

Radiofrequency energy was limited to 35 W along the LA posterior wall and roof regions and 50 W in the remaining areas, using institutional standard practice endpoints for ablation. All ablations were conducted in a point-by-point fashion on a power-controlled mode. Further radiofrequency ablation was delivered to achieve both near complete elimination (<0.1 mV) of local electrograms and elimination of positive vagal response following ablation at any site that demonstrated positive response during previous ablation attempt(s). A positive vagal response during radiofrequency application was defined as ≥20% decrease in sinus rate or ≥20% increase in PR interval. Additional right atrial ablation around the coronary sinus ostium for PMLGP and posteroseptal wall of SVC for RSGP was performed based on operator discretion.

The primary operators were classified according to their prior GP ablation experience: a single high-volume operator who had performed >50 GP ablation procedures (experienced operator), and operators performing their first GP ablation cases (first-time user).

### Extracardiac vagal stimulation

The use of extracardiac vagal stimulation (ECVS) was allowed based on the operator’s preference. The protocol of ECVS has been described previously.^15^ During ECVS, the vagus nerve was captured after placing a decapolar steerable electrode in the right or left internal jugular vein up to the level of the jugular foramen. Stimulation was delivered from the distal and the third pole of the electrode using pulsed electric field with a pulse amplitude of 1 V/kg of body weight up to 70 V, 50 ms width, and 30 Hz frequency, delivered over 5–8 s (neurostimulator designed by Pachon JCM, Sao Paulo, Brazil). Extracardiac vagal stimulation was performed from the right internal jugular vein and left internal jugular vein, respectively. The optimal site for pacing (reproducible sinus arrest and/or AVB) was obtained under fluoroscopic or ultrasonography guidance.

Acute procedural success was defined as follows: in patients with persistent resting AVB, achievement of 1:1 AV conduction; in patients with paroxysmal AVB, achievement of at least one of the following: 75% of the final PR interval that was recorded during pre-ablation atropine response test, a reduction in PR interval of >25%, or achievement of final sinus rate of <75% of that was recorded during the pre-ablation atropine response test.^9,14^

In the use of post-ablation ECVS, no changes in sinus rhythm and no AVB with proximal coronary sinus pacing [confidence interval (CI), 600 ms] were evaluated to predict procedural success.

### Follow-up

In all patients, clinical assessments and 12-lead ECGs were evaluated at discharge and at 1, 3, 6, and 12 months after ablation procedure. A prolonged ECG monitoring (24 h Holter, 72 h Holter, or implantable loop recorder) was evaluated at 3, 6, and 12 months and was repeated at >12 months in case of ongoing bradycardia-related symptoms. All patients were asked to record a diary of their symptoms, such as dizziness, fatigue, palpitation, or syncope episodes. No medical therapy was administered to patients except for post-procedural oral anticoagulation for at least 1 month. The primary outcome was freedom from syncope and any symptomatic (daytime) second-degree or higher degree AVB on Holter monitoring during follow-up.

### Statistical analysis

All statistical tests were performed with R version 4.3.2 (31 October 2023). Continuous data are presented as means (±standard deviation) or medians (interquartile range) as appropriate depending on the underlying distribution, and categorical data are summarized as frequencies and percentages. Wilcoxon rank sum test and Pearson's *χ*^2^ test were used as appropriate to evaluate differences in baseline characteristics. The cumulative risk of the primary study outcome over time was estimated using the Kaplan–Meier method. *P*-values <0.05 were considered as significant. Given the limited sample size, event number and, hence, statistical power of the presented data set, we conducted a least absolute shrinkage and selection operator (LASSO) regression in order to investigate which of the recorded covariables contributed most to the appearance of an event during the follow-up. A LASSO regression is a method allowing for both variable selection and regularization, which can be used when the number of variables approaches or exceeds the overall sample size; meaning in the case of high-dimensional data.^16^ It relies on penalization, meaning shrinking the coefficients of the least important variables to zero. The shrinkage allows for the reliable determination of the variables most predictive of the endpoints. To assess the performance of the model, the area under the curve (AUC) of the receiver operating characteristic curve was assessed.

## Results

### Baseline characteristics

A total of 130 patients [57% female; aged 34.00 (28.00, 44.75) years] were diagnosed as VAVB after screening with 12-lead ECG, 24 h Holter, exercise stress test, atropine testing, and EPS and underwent EACNA. Baseline characteristics are summarized in *Table 2*. In 14 (11%) cases, AVB was persistent before enrolment, of whom 7 patients had record of their persistent AV block captured on Holter and 1 on telemetry. In the remaining 116 (89%) patients, the AVB was paroxysmal and was identified on 24–72 h Holter monitoring in 90 patients, >7-day Holter in 8 patients, implantable loop recorder in 14 patients, and permanent pacemaker interrogation in 2 patients. In eight patients, paroxysmal atrial fibrillation was detected. A supra-Hisian level of block was confirmed by pre-ablation EPS in all persistent AVB cases. All patients, except for five patients, had at least one syncopal episode before enrolment.

**Table 2:** Baseline characteristics of patients with and without primary outcome

Characteristics	Overall (*n* = 130)	Primary outcome (+) (*n* = 17)^a^	Primary outcome (−) (*n* = 108)^a^	*P*-value^b^
Experienced operator (*n*, %)	67, 52	6, 35	59, 55	0.14
ECVS (*n*, %)	73, 56	7, 41	64, 59	0.2
Sex, female (*n*, %)	74, 57	8, 47	61, 56	0.5
Age (year)	34.0 (28, 45)	48.0 (33, 62)	34 (27, 42)	0.012
Number of syncope prior to enrolment	2 (1, 4)	1 (0, 4)	2 (1, 4)	
History of AF (*n*, %)	8, 6.2	5, 29	3, 2.8	0.001
Hypertension (*n*, %)	21, 16	9, 53	47, 44	<0.001
CAD (*n*, %)	5, 3.8	3, 18	2, 1.9	0.018
DM (*n*, %)	7, 5.4	1, 5.9	6, 5.6	>0.9
Heart rate (bpm)	62 (54, 70)	60	62.5	0.3
PR interval (ms)	180 (160, 210)	205.5 (180, 245)	180 (160, 200)	0.022
Paroxysmal AVB (*n*, %)	116, 89	13, 76	101, 95	0.021
Persistent AVB (*n*, %)	14, 11	4, 24	5, 4.7	

AF, atrial fibrillation; CAD, coronary artery disease; DM, diabetes mellitus; ECVS, extracardiac vagal stimulation; ILR, implantable loop recorder.

^a^Median (p25, p75) or frequency (%).

^b^Wilcoxon rank sum test and Pearson's χ^2^ test.

### Procedural characteristics

Acute procedural success was achieved in 125 (96.2%) of cases. The procedures took a median of 105.5 min (90–123 min). The CARTO mapping system (Biosense, Diamond Bar, CA, USA) was used in 77 patients and Ensite mapping system (Abbott, Saint Paul, MN, USA) was used in 63 patients. Most patients (90%) in the cohort underwent bi-atrial ablation. Of the 12 patients who did not undergo bi-atrial ablation, 5 patients underwent RA ablation only and 7 patients had LA only ablation based on operator preference. A median of 24 ablation points (18–35 points)^5,6,17–29^ were applied per patient. In all patients with accompanying atrial fibrillation, PV isolation was performed in addition to GP ablation. Procedural characteristics are summarized in *Table 3*. There were no major procedure-related complications.

**Table 3:** Procedural characteristics of patients with and without primary outcome

Characteristics	Overall (*n* = 130)	Primary outcome (+) (*n* = 17)^a^	Primary outcome (−) (*n* = 108)^a^	*P*-value^b^
Procedure duration (min)	105 (90–123)	120 (96–130)	105 (90–120)	0.2
3D mapping system, Carto (*n*, %)	77, 59	58	71	0.3
Anaesthesia, general (*n*, %)	94, 72	88	70	0.2
Bi-atrial ablation (%)	117, 90	94	90	>0.9
Total number of ablation points	24 (18–35)	24 (21–33)	24 (18–33)	0.7
Number of ablation points on LSGP	1.5 (0–4)	3 (1–4)	0 (0–4)	0.2
Number of ablation points on LIGP	3 (0–5)	3 (0–6)	2 (0–5)	0.5
Number of ablation points on MTGP	0 (0–4)	0 (0–3)	0 (0–4)	>0.9
Number of ablation points on RSGP	10 (7–15)	8 (4–11)	10 (8–15)	0.054
Number of ablation points on RIGP	0 (0–5)	2 (0–6)	0 (0–4)	0.2
Number of ablation points on PMLGP	8 (7–12)	8 (6–15)	8 (7–11)	0.9

3D, three-dimensional; LIGP, the inferior left atrial ganglionated plexus; LSGP, the superior left atrial ganglionated plexus; MTGP, the Marshall tract ganglionated plexus; PMLGP, the posteromedial left atrial ganglionated plexus; RIGP, inferior right atrial ganglionated plexus; RSGP, the superior right atrial ganglionated plexus. ^a^Median (p25, p75) or frequency (%). ^b^Wilcoxon rank sum test and Pearson's χ^2^ test.

Of the five patients for whom CNA was not deemed acutely successful, three patients had progression of AVB with ablation and two patients did not show improvement or resolution of AVB after GP ablation. Three of the five patients subsequently underwent permanent pacemaker implantation as a consequence, while two of the five patients already had a pacemaker.

### Follow-up

**Figure 1:**
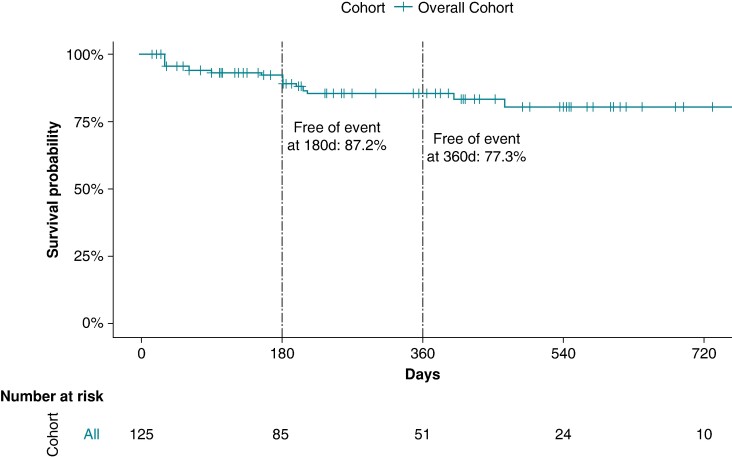
Survival analysis of the overall cohort with successful acute ablation.

All patients received post-procedural oral anticoagulation for at least 1 month with a non-vitamin K antagonist oral anticoagulant. The majority of patients (99/130, 76%) was followed using 24–72 h Holter monitoring. Over a median follow-up of 300 days (150–496 days, 76% with completed 180-day follow-up, 55% with completed 360-day follow-up), 14% of patients with acutely successful ablation (17/125) experienced an outcome event during the follow-up (8/17, 47% with syncope and 9/17 53% with second-degree or higher degree AVB). These events took place at a median of 152 days (30–196 days) post-ablation. When considering only patients with complete follow-up to this time point, 87.2% of the cohort (82/94) remained free of events at 180 days and 77.3% of the cohort (51/66) remained free of events at 360 days (*Figure 1*). In all patients who completed 360-day follow-up, three successive Holter recordings were evaluated for the final analysis during follow-up. Four of the nine patients who had primary outcome events required pacemaker placement because of syncope recurrence during follow-up after the index procedure. Three patients who experienced syncope during follow-up did not report history of syncope prior to their ablation procedures. These three patients complained of dizziness, fatigue, and >2 pre-syncope episodes. For one of these three patients, a correlation between the symptoms and transient AVB was established through Holter monitoring. Prior to undergoing CNA procedures and study enrolment, four patients had already undergone placement of permanent pacemakers but were still referred for CNA evaluation in hopes of being able to explant pacemakers due to patient age or because of pacemaker syndrome–mediated symptoms.

**Figure 2:**
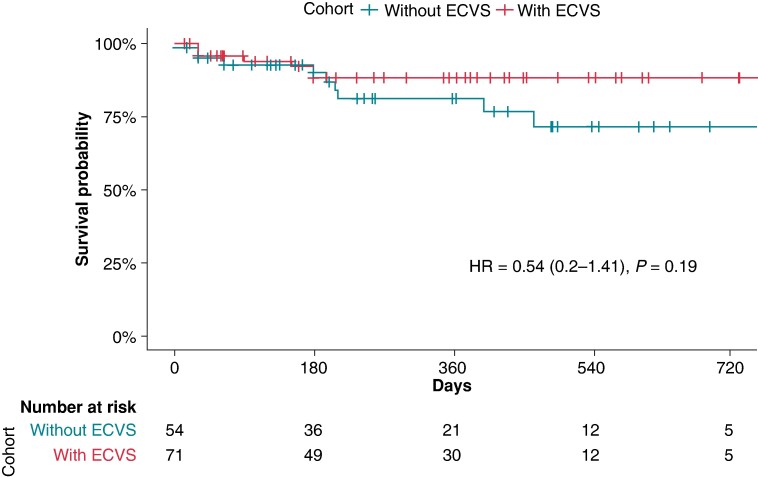
Survival analysis of the overall cohort with successful acute ablation depending on the use of extracardiac vagal stimulation. *P*-value given by the log-rank test for the full follow-up length. ECVS, extracardiac vagal stimulation; HR, hazard ratio.

**Figure 3:**
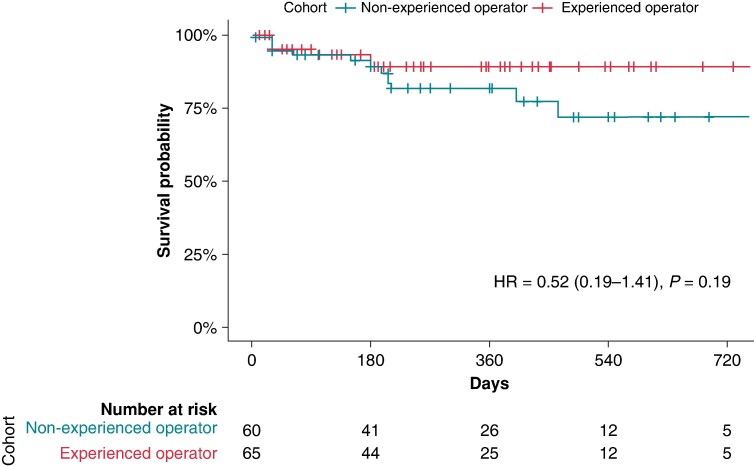
Survival analysis of the overall cohort with successful acute ablation depending on operator experience. *P*-value given by the log-rank test for the full follow-up length; HR, hazard ratio.

When analysing the cohort separately for the usage of ECVS (73/130, 56% with ECVS, 57/130, 44% without ECVS), there was no significant difference in primary outcome events of 10/54 (19%) in the group without ECVS vs. 7/71 (10%) in the group with ECVS, *P*-value_chi-square_ 0.2, *P*-value_log-rank_ = 0.19; *Figure 2*). Of the 125 successfully performed CNA procedures, 65 (52%) were performed by an experienced operator and 60 (48%) by a non-experienced operator. There was a non-significant trend for better outcome in the group ablated by an experienced operator [hazard ratio (HR) = 0.52, 95% CI (95% CI) 0.19–1.41, *P* = 0.19; *Figure 3*].

When the baseline characteristics are compared, persistent nature of AVB, older age, longer baseline PR interval, history of atrial fibrillation, hypertension, and coronary artery disease are found to be associated with primary outcome occurrence. When a LASSO regression was performed by inputting 10 variables (history of atrial fibrillation, hypertension, coronary artery disease, diabetes, PV isolation, baseline HR during the procedure, procedural duration, 3D mapping system used, type of anaesthesia, and conduction of bi-atrial ablation) in the model, 3 covariables (history of atrial fibrillation, hypertension, and coronary artery disease) were provided by the regression as most predictive of the outcome after regularization and shrinkage following cross-validation (*Table 4*). The model performed with an AUC of 0.74 (95% CI 0.61–0.87) to predict an event in the follow-up.

**Table 4:** Least absolute shrinkage and selection operator regression

Names	Estimate	Lower confidence interval	Upper confidence interval	*P*-value
History of atrial fibrillation	5.47	0.91	35.02	0.061
History of hypertension	4.82	1.24	17.63	0.018
History of coronary artery disease	3.14	0.32	33.74	0.319
Intercept	0.08	0.03	0.15	<0.001

## Discussion

The main findings of the current study are as follows: (i) acute procedural success can be achieved in 95% of patients by using EACNA; (ii) EACNA may reduce recurrences of syncopal episodes and second-degree or higher degree AVBs in patients with VAVBs during follow-up, obviating the need for a pacemaker; (iii) use of ECVS or operator experience did not have a significant role on ablation results; (iv) history of atrial fibrillation, hypertension, and coronary artery disease are associated with recurrence of syncope and second-degree or higher degree AVB.

The potential role of CNA in patients with AVB has been demonstrated in previous case series and single-observational studies.^3–12^ To the best of our knowledge, the present study is the largest multicentre study demonstrating the feasibility of EACNA with encouraging intermediate-term outcomes in appropriately selected patients with VAVB. In the current study, an electrogram-guided anatomical approach was used to map localization of GPs. The potential role of electrogram analysis for GP mapping has been first defined by using fast Fourier analysis requiring a computer-based software.^3^ Aksu *et al*.^29^ used visual evaluation of fractionated electrograms at filter settings between 200 and 500 Hz in electrophysiological recordings and demonstrated a similar improvement in clinical parameters with a shorter procedure time. The use of technique by first-time operators was compared with a single high-volume operator centre and demonstrated similar acute procedural and clinical success in an international multicentre study covering 16 centres.^17^ The present study confirms the usability of fractionated electrograms for GP mapping regardless of operator experience with a 95% acute procedural success rate in patients with VAVB.

In a recently published study including 115 patients with vasovagal syncope, intracardiac echocardiography was used to identify important anatomical landmarks and epicardial fat pads with presumed GP areas.^11^ Computed tomography–guided epicardial adipose tissue was also used in identifying GPs that were verified by high-frequency stimulation.^18^ In the present study, these two imaging methods were used in addition to electrogram analysis based on operator discretion.

To date, there are only case reports and small observational single-centre studies on patients with a combination of different vagally induced bradyarrhythmias, including vasovagal syncope, sinus bradycardia, and AVBs.^3–8,19–21^ In the only reported series of CNA that has focused on patients with VAVBs, 31 patients underwent EACNA (bi-atrial ablation in 90% of them), and their follow-up outcomes were evaluated with serial 24 h Holter monitoring.^9^ After a mean follow-up of 19.3 ± 15 months, 87.0% of cases were entirely free of syncope or documented AVB. In the current study, 77.3% of the cohort remained free of events for 360 days. As the most plausible explanation for lower event-free survival, long-term monitoring was used in 24% of the patients (>7-day Holter in 8 patients, implantable loop recorder in 14 patients, and permanent pacemaker in 2 patients), which may have increased the determination of the number of AVB episodes.

There is uncertainty regarding the best way to identify procedural end points during CNA procedures. Recently, to resolve the methodological problem of establishing procedural endpoints for CNA, which enables the assessment of vagal nerve influence on sinus and atrioventricular nodes (AVN), ECVS was suggested by Pachon-M *et al*.^22^ Although, in the present study, there was no significant difference in acute or clinical outcomes of CNA with and without ECVS, small number of recurrent cases can be the reason for this non-significant trend in favour of ECVS. Larger comparison studies are needed to answer this question. However, the use of ECVS was associated with overall longer procedure times. Extracardiac vagal stimulation evaluates the level of parasympathetic innervation, not damage to the epicardial ganglia or GP, to the AVN. Endocardial radiofrequency ablation in the immediate vicinity of the AVN may cause an axonal injury in post-ganglionic nerve fibres and may mimic acute parasympathetic denervation by post-ablation ECVS, which is defined as no changes in sinus rhythm and no AVB with proximal coronary sinus pacing. However, the effects on post-ganglionic nerve fibres might be short lived because axons can regenerate in healthy hearts. Hence, although no response to post-ablation ECVS seems like a reasonable endpoint for acute procedural success, this may not be related to clinical success during follow-up.

In the present study, persistent AVBs, older age, longer baseline PR interval, history of atrial fibrillation, hypertension, and coronary artery disease were associated with a higher primary outcome during follow-up. All these parameters might cause a structural disease of the AV conduction system and might be the possible reasons for only partial response to the CNA. In the present study, the risk factors for syncope recurrence were not further characterized due to the small number of cases. However, it is well known that recurrent syncope may predict increased mortality.^23^

### Study limitations

Several limitations of our study should be acknowledged. Although this is the largest published series evaluating the efficacy of CNA in patients with AVBs, the retrospective nature of the study is obvious, thereby offsetting the potential for selection bias because of a lack of knowledge about whether all consecutive patients from each centre were included. Any non-pharmacological measures or trigger avoidance was not attempted prior to inclusion. The quantitative burden of daytime symptoms caused by the AVBs was not reported by the centres. Thus, the efficacy of the procedure for the disappearance of symptoms cannot be reported in the present study. However, from a patient’s perspective the aim of ablation should be to improve symptoms rather than ‘curing’ an ECG. Also, baseline haemodynamic parameters such as heart rate and blood pressure, 24 h and office, were not reported by the centres. As one of the most serious complications of CNA, the incidence, timing, and resolution of iatrogenic inappropriate sinus tachycardia were not uniformly assessed by the operators, which may worsen the quality of life and require heart rate controlling drugs, such as ivabradine and beta-blockers.

Due to the low number of events on follow-up, we could not separately perform subanalyses to meaningfully define differences in characteristics between patients who had syncope and those with symptomatic AVB recurrences without syncope. A more recent workup proposal includes tilt testing and ambulatory blood pressure monitoring for evaluation of presumable vagally induced syncope, which was not performed in the present study.^24–26^ An increase in average 24 h systolic blood pressure, regardless of the implemented strategy, significantly reduced symptom burden.^27^

The differences in procedural techniques and in assessing acute procedural success (ECVS vs. non-ECVS) are other limitations of the present study. There was no control group, and hence, the potential for a placebo/expectation effect cannot be ruled out totally; however, we used strict objective criteria to assess ablation efficacy during the procedure and deployed serial Holter ECG monitoring to document the absence of AVB on follow-up. Until a randomized controlled trial is completed, the results of this study should be taken with caution. Intermediate-term follow-up is another limitation because the long-term clinical outcome of CNA may be affected by consequent reinnervation by failure to eliminate epicardial ganglia. It will be necessary to continue follow-up to see whether the results are sustained. Also, prolonged ECG monitoring by event recorder, implantable loop recorder, or cardiac pacing devices was only employed in a minority of the present group, which may have underestimated the recurrence rate of AVB.

In the present study, fractionated electrograms were used to map localization of GPs in a young population with healthy atria. However, the presence of fractionated atrial electrograms may be less specific for GP sites in the presence of a diseased myocardium (e.g. fibrosis) or heterogeneous myocardial conduction. In a recently published study, computed tomography–guided identification of epicardial adipose tissue was used to map GPs.^28^ Further research is needed to define the best GP mapping strategy.

## Conclusions

The present multicentre study demonstrated the feasibility of EACNA with encouraging intermediate-term outcomes in highly selected patients with VAVBs. Although these findings are promising, randomized controlled trials with longer term follow-ups are needed to assess the efficacy and durability of the technique.

## Data Availability

All relevant data are within the manuscript.
